# Fatigue Life Appraisal and Its Corrected Stress Intensity Factor for Repaired Off-CentrallyCracked Aluminum Plates

**DOI:** 10.3390/ma13184014

**Published:** 2020-09-10

**Authors:** Xiang You, Zhiyu Wang, Xiafang Zhou, Zifeng Liu, Ruijuan Jiang, Weiming Gai

**Affiliations:** 1 Key Laboratory of Deep Underground Science and Engineering (Ministry of Education), School of Architecture and Environment, Sichuan University, Chengdu 610065, China; you_flying@sina.com (X.Y.); 18054023500@139.com (X.Z.); 17761283846@139.com (Z.L.); 2 Sichuan Provincial Key Laboratory of Failure Mechanics and Engineering Disaster Prevention & Mitigation, Sichuan University, Chengdu 610065, China; 3 Innovation Design Department, Shenzhen Municipal Engineering Design and Research Institute Co. Ltd., Shenzhen 518029, China; gaiwm@szmedi.com.cn

**Keywords:** off-central crack, fatigue life, stress intensity factor, fracture mechanics

## Abstract

This paper presents an experimental study on the fatigue life estimation of off-centrally cracked aluminum plates. Typical theoretical equations for off-central, central and edge cracks were reviewed and compared in terms of their sensitive parameters and applicability. A finite element model has been validated in its capacity in modelling the influences of eccentricity and crack size on the boundary correction coefficients. The Forman equation has been employed along with numerical results for the prediction of fatigue lives. Based on the test data, the fatigue life results of aluminum plates with and without patched laminate repair have been compared with codified fatigue classes. It is demonstrated that the repair at the crack tip close to the plate edge is effective in the fatigue life improvement for off-centrally crackedaluminum plates.

## 1. Introduction

**Figure 1:**
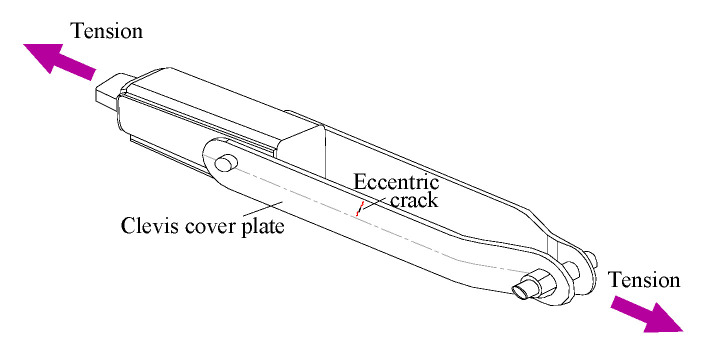
Off-central crack in a typical tension clamp.

Tension structural components, such as tension clamps made up of clevis cover plates in tension, play an important role in many railway [1,2] or aircraft [3,4] mechanical systems. Using codified variables, it is relatively easy to find a premade tension structural component to suit the specifications of a specific project. Fatigue cracks in a tension structural component usually initiate at notches as a result of stress concentration. Since the fatigue cracks can start very early within the fatigue endurance, the fatigue life estimation becomes a major question for the fatigue crack propagation analysis. For such a purpose, the stress intensity factors for a central crack or an edge crack as typical crack formations have been intensively investigated by contemporary researchers and documented in design manuals, such as Reference [5]. During services, however, mechanical failure can take place, due to the formation of cracks at the initial imperfections or flaws away from the center or the edge. An example in Figure 1 indicates an offset imperfection-related fatigue crack initiated between the edge and the center line of the clevis cover plate subject to tension.

The linear elastic fracture mechanics method has gained a good application for the prediction of the strength and life of cracked structures, when the understanding of the crack tip stress intensity factor as a function of applied load and structural geometry is known. The crack tip can be assumed as an energy sink, around which dissipation takes place for energy propagation. The research work reported by Isida [6] seems to give the first theoretical equation in the prediction of stress intensity factors for the tension of an eccentrically cracked strip. The Airy stress function for a state of generalized plane stress can be formulated in terms of complex potentials, which can be expanded in Laurent’s series, convergent in a region bounded by two certain concentric circles. The resultant stress intensity factors are given in power series, and their related coefficients are tabulated. Further understanding of the stress intensity factor of eccentrically cracked plateshas deepened in theory in the past two decades. For example, Wang [7] proposed a quite useful, simple theoretical expression with the balance of force and moment for the above-mentioned stress intensity factor, which results in a good correlation with Isida’s report [6] with less than 6% error. Following Isida’s report [4], Wang et al. [8] recently developed means based on the Westergaard stress function, which is simplified to provide solutions for the crack opening displacement and stress-strain field ahead of an eccentric crack. The length from the maximum crack opening site to the crack tip was suggested as the length of the crack when the Westergaard stress distribution is concerned. To validate the theoretical findings, numerical modelling has also been performed by researchers in the study of stress intensity factors of eccentrically cracked plates. For example, Wang et al. [9] adopted a macro element method in the calculation of stress intensity factors and analysis of cracked plate. It was shown that the crack propagation may become stable when the eccentricity is considered. Later, the detrimental effect of the presence of offset cracks on the tension plate with eccentric cracks embedded in bi-materials was observed by Ismail [10], since the formation of mixed mode stress intensity factors at the crack tip lead to premature failure. Subsequently, Han et al. [11] compared and discussed the efficiencies of the displacement extrapolation method, stress extrapolation method, node displacement method, and J-integral method based on finite element analyses of stress intensity factors of eccentrically cracked plates. Triangular singular elements, and an element size of 1/10 of the crack length, are suggested for increased accuracy in the prediction of stress intensity factors. Recently, an equivalent model was developed by He et al. [12] for an eccentrically cracked plate with clamped ends. Experimental data waste was avoided using approximate solutions, since invalid crack growth data, failing to meet the crack symmetry condition, could be utilized in the data processing.

Where cracking occurs in aluminum plates, a satisfactory repair may be made by composite patches and adhesive bonding in the reinforcement. Their attractive merits are good flexibility, high strength-to-weight ratio, and good capacity in resisting environment deterioration or corrosion. Ayatollahi and Hashemi [13] conducted finite element modelling in the study of the effect of composite patching, such as composite laminate configuration, and adhesive properties on the crack tip parameters. Related stress intensity factors along with the fracture strength of the repaired specimens under mixed mode loading conditions were analyzed. Khan et al. [14] performed fatigue tests on V-notched repaired and unrepaired pre-cracked specimens under fatigue loading. It was found that very high fatigue life extension can be achieved when the patch precedes the overload, which can be owed to a cumulative retardation effect of patching and overload. Recently, the authors have investigated the effect of CFRP (carbon fiber reinforced polymer) reinforcement on the fatigue strength of corrugated plates [15,16], steel plates [17,18], and aluminum alloy plates [19,20,21,22,23,24,25] with central fastener holes. Based on the test and modelling data, a new model has been proposed for the contributions of the plate and carbon fiber in tension, the adhesive interface in shear, and additional secondary bending effect when the improvement fatigue endurance is concerned.

Despite the above-reviewed research work, there are no unanimous conclusions for the stress intensity factors of off-centrally cracked aluminum plates, with and without patched laminate repair and subject to fatigue loading. In this paper, the theoretical equations in the calculation of the stress intensity factors for off-centrally cracked aluminum plates are compared. A counterpart three-dimensional finite element model is developed for the stress intensity factor calculation and validated with the calculation results of cracked aluminum plates without strengthening. Based on the fatigue test results, the improvement of fatigue life of the patched, laminate-repaired, off-centrally cracked aluminum plate is discussed with codified curves.

## 2. Experimental Procedure and Results

**Figure 2:**
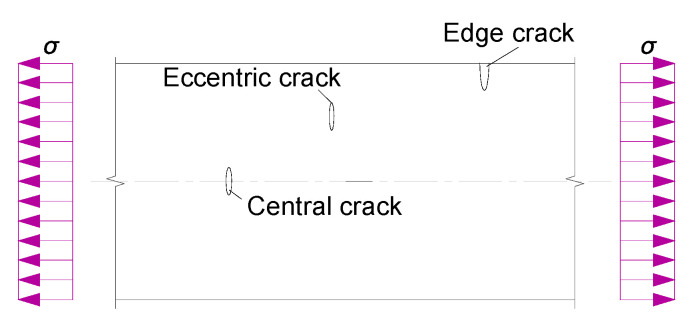
Crack modes in a tension strip.

**Figure 3:**
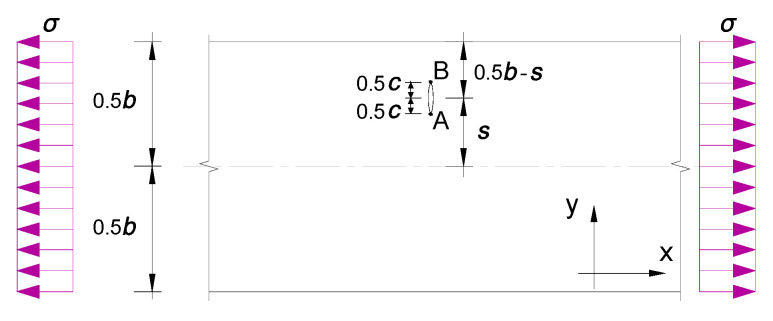
Analytical illustration of an off-centrally cracked strip.

**Figure 4:**
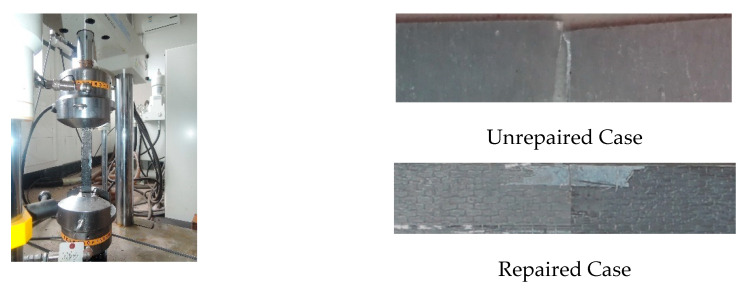
Test set-up and fracture mode.

Test specimens were machined from aluminum sheet (Al 2024T3) (Alcoa Korea, Ltd., Seoul, Korea), satisfying the American specification AMS-QQ-A-250/5A. The specimen plates were 300 mm long by *b* = 30 mm wide by *t* = 2 mm thick. The off-central crack was considered between the centerline and the tip of the plate, which can be regarded at an intermediate location between the central crack and edge crack, as shown in Figure 2. Before patched laminate repair, the specimen plates were notched through the thickness of the crack started on both sides (tip A and tip B), for which the total initial crack length and the eccentricity are denoted as *c* and *s*, respectively, as shown in Figure 3. The patched laminates were made using unidirectional CFRP (carbon fiber reinforced polymer) (Toray Group Co., Ltd., Tokyo, Japan) applied at one side of test aluminum alloy plate in alignment with the longitudinal direction (x direction) of the test specimen. The CFRP was cured and bonded to the pre-cracked specimen using epoxy resin matrix Sikadur 330CN (Sika Corporation, Lyndhurst, NJ, USA). As listed in Table 1, the mechanical property of the aluminum sheet was obtained from monotonic tensile tests of six coupons(Toray Group Co., Ltd., Tokyo, Japan) under the loading rate of 0.02 mm per second, while that of the carbon fiber and epoxy resin matrix were obtained according to GB50367 and DIN 53,455,respectively, referring to the testing reports from the manufacturers. In total, 21 specimens grouped in the four series (“centrally cracked + unrepaired”, “centrally cracked + repaired”, “off-centrally cracked + unrepaired” and “off-centrally cracked + repaired”) were tested with a varying eccentricity ratio of 2*s*/*b* and the crack size ratio of *c*/*b* as listed in Table 2. Fatigue tests were conducted using a servo-hydraulic testing machine (Shimadzu, Kyoto, Japan), as shown in Figure 4, with the test frequency of 8 Hz until the fracture of the test specimen. As tress ratio of *R* = 0.1 and maximum stress range of 170 MPa sinusoidal waveform was used to fatigue specimens. Repetitive load signals were measured using a PC-based automatic data acquisition system.

## 3. Finite Element Analysis

The commercial finite element modelling package ANSYS software (ANSYS Inc., Canonsburg, PA, USA) was employed to numerically simulate the stress distribution in front of both crack tips, and the stress intensity factors for the off-central cracks. The modelling results were adopted to validate the results calculated from referred theoretical equations, which will be presented in the following section. The material mechanical properties in the finite element models are the same as those in the experimental tests, as listed in Table 1.

**Figure 5:**
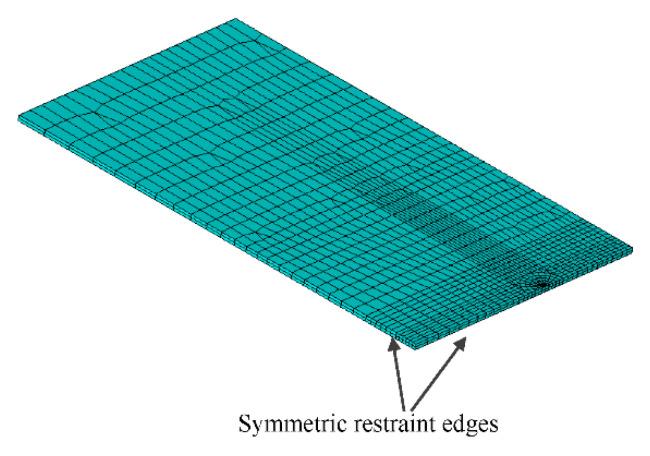
Typical FE model.

Since the test specimens are shown in Figure 4 to rupture perpendicular to the longitudinal direction of the test plate with slight necking on the plate fringe, only the upper half of the test specimen was modelled due to the symmetry condition before and after the rupture of the test specimen, as shown in Figure 5. As such, the bottom two edges except the crack line between two crack tips are symmetrically restrained, while the longitudinal tensile stresses were applied at the tip edge.

**Figure 6:**
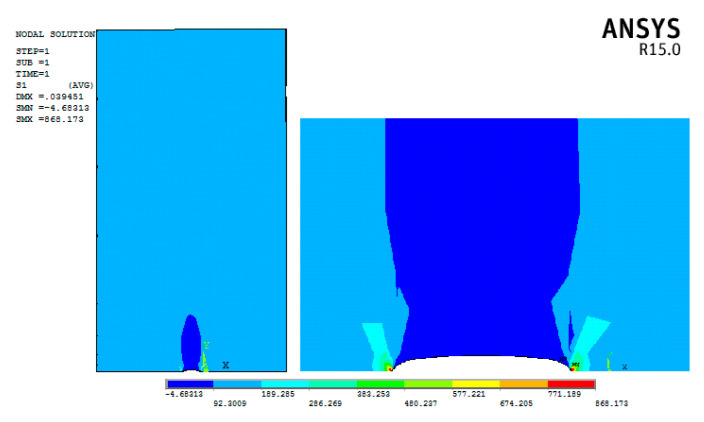
Stress concentration at crack tip.

This failure is expected, as the maximum tensile stress in the vicinity of the crack tip B is close to the edge of the plate, which agrees well with the modelling results as shown in Figure 6. The J-integral calculation technique with a square root singularity at the crack tip was applied for the analysis of the stress intensity factor at the crack tip. The quadratic solid elements SOLID95 were used in the mesh construction of the crack tip zone with a swept meshing method. Only the meshes near the crack tip zone were intensified with the element size of 0.5 mm due to the consideration of computational costs. For the geometry of the finite element model, the eccentricity ratio of 2*s*/*b* and the crack size ratio of *c*/*b* are varied as 0.17~0.7 and 0.05~0.5, respectively, so as not to surpass the plate width and contradict the cases related to central or edge cracks.

## 4. Results and Discussion

### 4.1. Summary of K_I_ and F_w_ Theoretical Equations for Off-Central, Central and Edge Cracks

Based on the theory of fracture mechanics, the mode I elastic stress intensity factor can be assessed by the following criteria:

(1) $$K_{I}=F_{W}\sigma \sqrt{0.5\pi c}$$

which is determined by the nominal tension stress, *σ*, and the boundary correction coefficient, *F*_w_. For the surface crack scenario studied herein, suitable parameters are needed for the rational assessment of *F_w_*. On this basis, two analytical equations are compared as below.

#### 4.1.1. Crack Line Stress Field Method for Off-Centrally Cracked Case

Based on the model proposed by Wang [5], a crack extended line subject to uniaxial tensile stress *σ* consists of *K*_I_ dominated plastic segments at the crack tips (A and B) and outside plastic segments. Based on the basic theory for a crack opening symmetrically with respect to the undeformed crack plane, the corresponding normal stress *σ*_x_ along the *x* axis is written as:

(2) $$\sigma_{x}=\left\{ \begin{array}{l} \frac{F_{\mathrm{WA}}\sigma \sqrt{0.5\pi c}}{\sqrt{2\pi 0.5d}}=\frac{F_{\mathrm{WA}}\sigma \sqrt{c}}{\sqrt{2d}}\;(0<0.5d\leq 0.5d_{0A})\;\\ =\frac{F_{\mathrm{WB}}\sigma \sqrt{c}}{\sqrt{2d}}\;(0<0.5d\leq 0.5d_{0B}) \end{array}\right.$$

where, *F*_WA_ and *F*_WB_ are the correction factors, *d* is the diameter of pseudo plastic zone at the crack front. When the distances from the centers of the pseudoplastic zone are greater than 0.5*d*_0A_ or 0.5*d*_0B_, the square *F*_WA_ or *F_WB_* is chosen due to an allowance of finite width and the corresponding normal stress *σ*_x_ is expressed as:

(3) $$\sigma_{x}=\left\{ \begin{array}{l} F_{\mathrm{WA}}^{2}\sigma \;(0.5d_{0A}\leq 0.5d\leq 0.5b+s-0.5c)\\ F_{\mathrm{WB}}^{2}\sigma \;(0.5d_{0B}\leq 0.5d\leq 0.5b-s-0.5c) \end{array}\right.$$

Given the stress continuity at the boundary of the plastic zone around the crack origin, i.e., Equation (2) equal to Equation (3) when *d = d*_0__A_ and *d = d*_02__B_, it can be obtained as *d*_0__A =_ 0.5*c*/*F*_WA_^2^ and *d*_0__B =_ 0.5*c*/*F*_WB_^2^. Equating the tensile stress along the crack extended line to the remote tension stress, *σ*, along the *y* axis yields:

(4) $$\int_{0}^{0.5d_{0A}}\frac{F_{\mathrm{WA}}\sigma \sqrt{c}}{\sqrt{2d}}\partial d+\int_{0}^{0.5d_{0B}}\frac{F_{\mathrm{WB}}\sigma \sqrt{c}}{\sqrt{2d}}\;\partial d+F_{\mathrm{WA}}^{2}\sigma (0.5b+s-0.5d_{0A}-0.5c)+F_{\mathrm{WB}}^{2}\sigma (0.5b+s-0.5d_{0B}-0.5c)=b\sigma$$

In equilibrium along the crack extended line, the sum of bending moments about the centerline of the tubular flange is zero, and thus:

(5) $$\begin{array}{l} \int_{0}^{0.5d_{0A}}\frac{F_{\mathrm{WA}}\sigma \sqrt{c}}{\sqrt{2d}}\partial d(0.5d_{0A}+0.5c-s)+0.5F_{\mathrm{WA}}^{2}\sigma \left[0.25b_{}^{2}-{\left(0.5d_{0A}+0.5c-s\right)}^{2}\right]\\ =\int_{0}^{0.5d_{0B}}\frac{F_{\mathrm{WB}}\sigma \sqrt{c}}{\sqrt{2d}}\partial d(0.5d_{0B}+0.5c+s)+0.5F_{\mathrm{WB}}^{2}\sigma \left[0.25b_{}^{2}-{(0.5d_{0B}+0.5c+s)}^{2}\right] \end{array}$$

Given the (1/*F*_WA_^2^-1/*F*_WB_^2^) approaching zero when *s =* 0 (no eccentricity), solving Equations (4) and (5) gives:

(6) $$F_{\mathrm{WA}}=\sqrt{\frac{2b+4s-c}{2b+4s-2c}}$$

(7) $$F_{\mathrm{WB}}=\sqrt{\frac{2b-4s-c}{2b-4s-2c}}$$

#### 4.1.2. The Westergaard Function Based Method for Off-Centrally Cracked Case

The stress distribution in front of the crack tip along crack line can be expressed using the Westergaard function as:

(8) $$\sigma_{x}=\frac{F_{W}}{\sqrt{1-{\left(\frac{0.5c}{x}\right)}^{2}}}$$

where, *x* is the distance from the crack center in the crack line. At two sides of the crack (close to crack tip A and B), the corresponding loads carried by the stress distributions at uncracked parts are given by:

(9) $$P_{A}=\int_{0.5c}^{0.5b_{0}+s-0.5c}\sigma_{x}t\partial y$$

(10) $$P_{B}=\int_{0.5c}^{0.5b_{0}-s}\sigma_{x}t\partial y$$

The stress distributions far away from the cracked section are given by:

(11) $$P_{A}=\sigma t(0.5b_{0}+s-0.5c)$$

(12) $$P_{B}=\sigma t(0.5b_{0}-s)$$

Implementing the load equilibrium Equation (9) = Equation (11), and Equation (10) = Equation (12) at the cracked section, the boundary correction coefficient developed by Wang et al. [6] is written as:

(13) $$F_{\mathrm{WA}}=\frac{1}{\sqrt{1-{\left(\frac{c}{b+2s-c}\right)}^{2}}}$$

(14) $$F_{\mathrm{WB}}=\frac{1}{\sqrt{1-{\left(\frac{c}{b-2s}\right)}^{2}}}$$

#### 4.1.3. Comparison of Referred Calculations]

**Figure 7:**
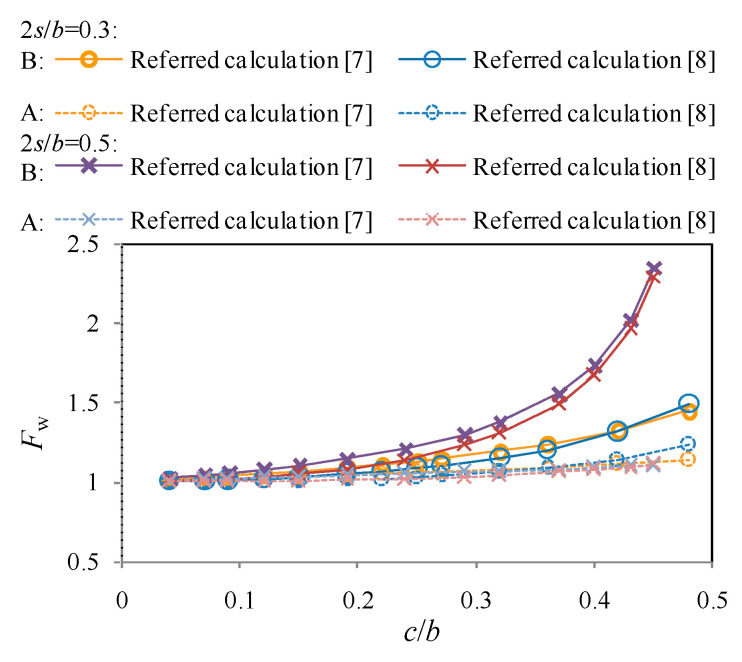
Comparison of referred calculations of stress intensity factors (SIF) at A and B.

In Figure 7, the boundary correction coefficients at the crack tips for off-centrally cracked plates calculated using referred methods are compared against the crack size ratio of *c*/*b*. The results obtained from the crack line stress field method are slightly higher than the Westergaard function-based method, especially when *c*/*b* is ranging between 0.2 and 0.35. However, such a difference is gradually reduced as *c*/*b* is greater than 0.4. With the increasein the eccentricity, calculated *F*_w_ is increased which indicates a much higher stress concentration of the stress field at the crack tip. Calculated *F*_w_ for crack tip A is consistently lower than that for crack tipB and seemingly less influenced by the eccentricity of the crack. Such an underestimation can be owed to the different plastic zones at the crack fronts of tip A and B, which was simplified as the same in referred methods. For design purpose, however, only the larger *F*_w_, i.e., for crack tip B, related to greater stress concentration is considered in the following analysis.

### 4.2. Finite Element Analysis Based Evaluation of F_w_ for Off-Centrally Cracked Plates

Given that the aforementioned theoretical methods are limited by simplified assumptions, *F*_w_ for off-centrally cracked plates is evaluated based on a finite element parametric study. One hundred and twelve finite element models—which are permutations and combinations of seven sets of 2*s*/*b* ranging from 0.17 to 0.7, and fourteen sets of *c*/*b* ranging from 0.1 to 0.5—are built for the stress intensity factor analysis. Based on the finite element parametric study, the calculation for *F*_w_ can be developed as the following poly-fit equation, including the ratios of 2*s*/*b* and *c*/*b*.

(15) $$F_{\mathrm{WB}}=\left[212{\left(\frac{2s}{b}\right)}^{3}-173{\left(\frac{2s}{b}\right)}^{2}+53\left(\frac{2s}{b}\right)-3.7\right]{\left(\frac{c}{b}\right)}^{2}-0.05e^{5.5\left(\frac{2s}{b}\right)}\left(\frac{c}{b}\right)+e^{0.12\left(\frac{2s}{b}\right)}$$

**Figure 8:**
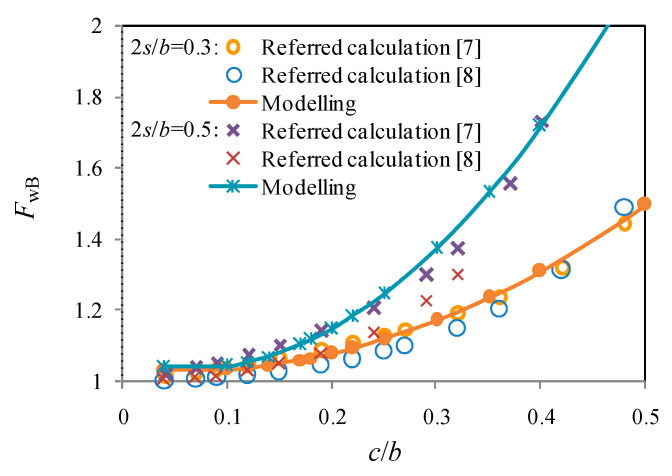
Off-central crack in tension clamp.

As a validation example for the cases of 2*s*/*b =* 0.3 and 0.5 shown in Figure 8, *F*_w_ predicted from modelling agrees well with those from referred calculation. The cases with central cracks and edge cracks are also referred to further the comparison with analytical results. For the centrically cracked plate of finite width, the stress intensity factor at the crack tip was originally modified from that for an infinite sheet with a periodic array of through-thickness cracks under uniformly distributed stress. The formation is deemed accurate for up to *c*/*b =* 0.5, and the tangent correction as documented in Reference [3] is given by:

(16) $$F_{W}=\sqrt{\frac{2b}{\pi c}\tan\frac{\pi c}{2b}}$$

An alternative solution was later presented by Feddersen [26], and known as the secant correction, as:

(17) $$F_{W}=\sqrt{\sec\frac{\pi c}{2b}}$$

The stress intensity factor at the crack tip for the edge cracked plate was originally modified from the long surface crack solution when in-plane bending was not accounted. The corresponding boundary correction coefficient documented in BS 7910 [27] is:

(18) $$F_{W}=1.12-0.23\left(\frac{c}{b}\right)+10.6{\left(\frac{c}{b}\right)}^{2}-21.7{\left(\frac{c}{b}\right)}^{3}+30.4{\left(\frac{c}{b}\right)}^{4}$$

**Figure 9:**
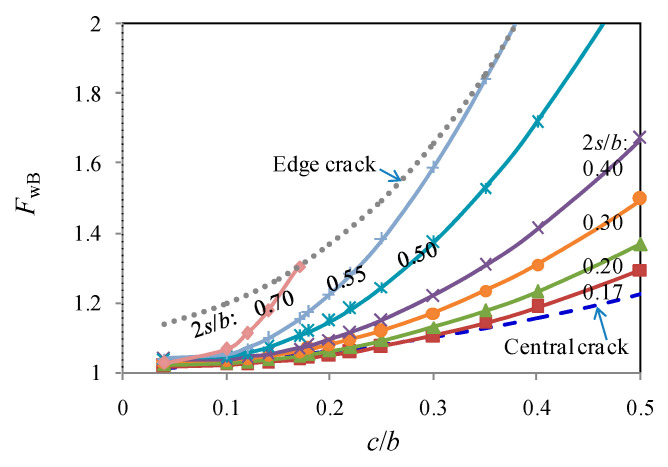
Crack modes in a tension strip.

As observed from Figure 9, the curves predicted from the above poly-fit equation are governed by off-central cracks (i.e., within the range between those for the edge crack and those for the central crack) when 2*s*/*b* is increased from 0.17 to 0.55. Conversely, *F*_w_ is more or less involved with the edge cracks or the central cracks when the 2*s*/*b* is greater than 0.55 or less than 0.17.

### 4.3. Fatigue Life Evaluation

**Figure 10:**
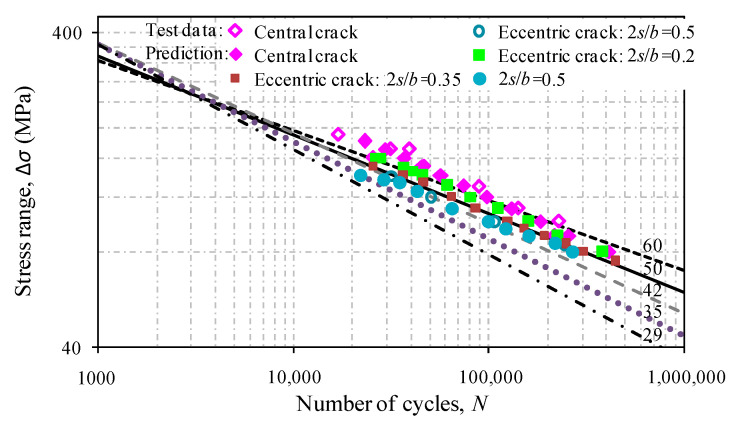
Comparison of fatigue live results between centrally and off-centrally cracked plates.

Using the above-discussed boundary correction coefficient, *F*_W_, and the mode I elastic stress intensity factor, the fatigue life of the test specimens can be evaluated. As per the suggestion by Su et al. [28], the aluminum material was defined following a rate-independent isotropic elastic-plastic relation using the classical *J*_2_-flow theory of the plasticity. It fits the experimentally measured stress-strain curve with the extraction of strain-hardening features. When above discussed boundary correction coefficient, *F*_W_, is determined, the mode I elastic stress intensity factor can be calculated using Equation (1), and then the fatigue life of test specimens can be evaluated. It is generally recognized that three regions exist for the relationship between the fatigue crack growth rate and the stress intensity factor; threshold, linear growth, and accelerated growth. To calculate the fatigue life, the crack propagation during the latter two regions can be analyzed using the concept of fracture mechanics [29] and the well-known Forman equation [3], including the stress ratio effect and *F*_W_ aforementioned in the Section 4.2, as:

(19) $$\frac{dc}{dN}=\frac{C_{F}{\left(\Delta K_{I}\right)}^{m}}{(1-R)K_{C}-\Delta K_{I}}=\frac{C_{F}{\left(F_{W}\Delta \sigma \right)}^{m}{\left(0.5\pi c\right)}^{0.5m}}{(1-R)K_{C}-F_{W}\Delta \sigma \sqrt{0.5\pi c}}$$

where, *K*_c_ is the fracture toughness of the test material, which is equal to 1.181 × 10^3^ MPa·mm^0.5^. *C*_F_ is the material constant converted from its counterpart in Paris equation as 0.174 × 10^−10^. Assuming *m =* 3, the integration of Equation (19) results in the fatigue life, *N*, of the cracked aluminum plate as:

(20) $$N=\frac{2(1-R)K_{C}(\sqrt{c_{0}}-\sqrt{c_{1}})}{C_{F}{\left(F_{W}\sqrt{\pi }\Delta \sigma \right)}^{3}}-\frac{1}{C_{F}{\left(F_{W}\sqrt{\pi }\Delta \sigma \right)}^{2}}\ln\frac{c_{1}}{c_{0}}$$

where, *c*_0_ and *c*_1_ are the initial and final crack lengths, respectively. As the magnitude of Δ*K*_I_ is determined by *c*_0_ and *c*_1_, *N* can be obtained via the numerical analysis and corresponding data for *S*-*N* relation can be deduced. Figure 10 shows a good correlation of fatigue lives between experimental test results and finite element modelling based analytical results using the Forman equation when the centrally cracked and off-centrally cracked aluminum plates are concerned. This also indicates that *F*_w_ defined by the proposed poly-fit equation would be a good choice in representing stress intensity factors, and thus the fatigue life.

Based on the fatigue criterion recommended by the BS8118 standard for structural use of aluminum [30], the fatigue test data and prediction results are compared against detail classes. As shown in Figure 10, codified *S*-*N* curves are represented as the fatigue strength levels at 2 million cycles. The data of logΔ*σ*-log*N* calculated for off-centrally cracked plates with 2*s*/*b =* 0.2 are slightly lower than those for centrally cracked plates over the classes 50 and 60. In contrast, the test data for off-centrally cracked plates with 2*s*/*b =* 0.5 are shown to fall above the class of 35. Despite the scatter of the predictions, a consistent trend can be observed for the off-centrally cracked plate with a listed variation of 2*s*/*b.*

**Figure 11:**
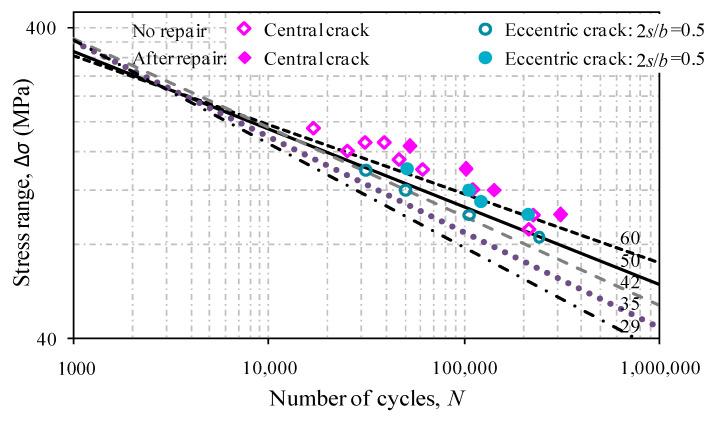
Comparison of fatigue live results between unrepaired and repaired plates.

**Figure 12:**
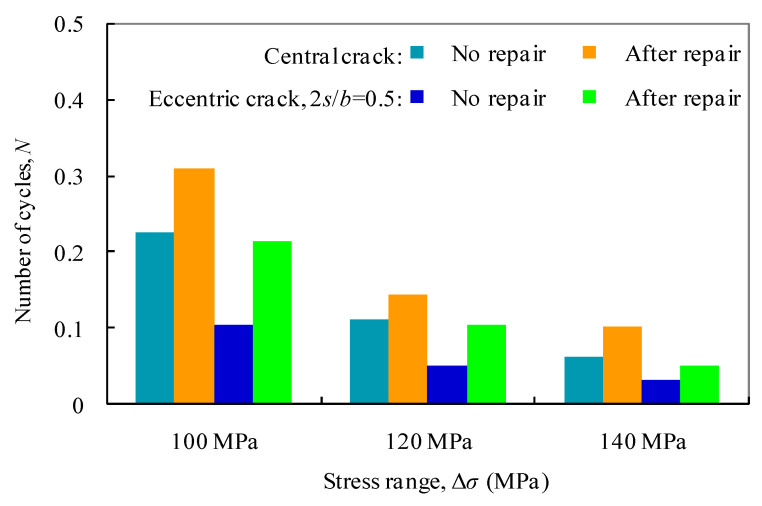
Comparison of fatigue lives of cracked plates under three typical stress ranges.

The comparison of *S*-*N* curves for test aluminum plates with and without patched laminate repair is shown in Figure 11. The test fatigue lives under stress ranges of 100, 120, and 140 MPa are extracted for the comparison in Figure 12. After repair, it can be seen that the fatigue lives of the test specimens with central cracks are shown to increase by 30–60%. This can be due to the beneficial effect of the carbon fiber and interface strengthening, and the decrease in stress concentration at thecentral crack hole. In contrast, such a beneficial effect becomes more effective for off-centrally cracked plates with 2*s*/*b =* 0.5, for which the fatigue lives are increased by 90–120%, as a result of repair especially at the crack tipB, close to the plate edge in Figure 3.

## 5. Concluding Remarks

The stress intensity factors for off-centrally cracked aluminum plates have been presented in this paper. Typical theoretical equations for off-central, central and edge cracks were reviewed and compared in terms of their sensitive parameters and applicability. A finite element model has been developed to explore a wide range of parameters related to the eccentricity and crack size influencing the boundary correction coefficients. The Forman equation has been employed, along with numerical results for the prediction of fatigue lives. Based on the test data, the fatigue lives result of aluminum plates with and without patched laminate repair have been compared with codified fatigue classes. The efficiency of repair for the improvement of fatigue lives is compared and discussed for central and off-central cracked conditions. The following conclusions can be drawn:

- The developed finite element parametric study-based poly-fit equation for the boundary correction coefficient incorporating the eccentricity ratio and the crack size ratio is demonstrated to agree well with referred calculations. Moreover, it fits in well between centrally cracked cases and edge crack casesfor the off-centrally cracked aluminum plates.
- The fatigue life prediction on the basis ofthe Forman equation accounting for the crack in linear growth and accelerated growth is shown to correlate well with the test results of aluminum plates with central cracks and off-central cracks. The corresponding *S*-*N* curves are also comparable to those suggested by codified curves.
- The beneficial effect of patched laminate repair can be identified from the increase in test fatigue lives by 30–60% and 90–120% for centrally cracked and off-centrally cracked aluminum plates, respectively. Thus, the repair at the crack tip close to the plate edge is deemed to be effective in the fatigue life improvement for off-centrally crack aluminum plates.The strengthening effect of patched laminate repair, however, was not modelled in detail, and therefore requires more experimental work in furthering the correction of defined parameters in the fatigue life prediction.

## Figures and Tables

**Table 1:** Mechanical properties of test materials.

Material	Yield Strength (MPa)	Ultimate Strength (MPa)	Elastic Modulus (MPa)	Elongation (%)	Critical Stress Intensity Factor (MPa·mm^0.5^)
Aluminum	307	445	7.2 × 10^5^	15	1181
Carbon Fiber	-	4216	2.52 × 10^5^	1.76	-
Adhesive	-	30	4.5 × 10^3^	0.9	-

**Table 2:** List of test specimens.

Specimen Series	Test Number	Crack Mode	Repair	2*s*/*b*	*c*/*b*
T1	9	Centrally	×	0	0.15–0.45
T2	4	Centrally	√	0	0.3
T3	4	Off-Centrally	×	0.5	0.3
T4	4	Off-Centrally	√	0.5	0.3
